# Health system governance to support scale up of mental health care in Ethiopia: a qualitative study

**DOI:** 10.1186/s13033-017-0144-4

**Published:** 2017-06-08

**Authors:** Charlotte Hanlon, Tigist Eshetu, Daniel Alemayehu, Abebaw Fekadu, Maya Semrau, Graham Thornicroft, Fred Kigozi, Debra Leigh Marais, Inge Petersen, Atalay Alem

**Affiliations:** 1 Department of Psychiatry, School of Medicine, College of Health Sciences, Addis Ababa University, Addis Ababa, Ethiopia; 2 Centre for Global Mental Health, Health Services and Population Research Department, Institute of Psychiatry, Psychology and Neuroscience, King’s College London, London, UK; 3 Department of Psychological Medicine, Centre for Affective Disorders, Institute of Psychiatry, Psychology and Neuroscience, King’s College London, London, UK; 4 Butabika National Referral and Teaching Hospital, Makerere University, Kampala, Uganda; 5 Research Development & Support Division, Faculty of Medicine & Health Sciences, Stellenbosch University, Cape Town, South Africa; 6 School of Nursing and Public Health, University of KwaZulu-Natal, Durban, South Africa; 7 School of Applied Social Sciences, University of KwaZulu-Natal, Durban, South Africa

**Keywords:** Governance, Systems thinking, Health systems, Developing country, Mental health services, Mental health policy, Primary care

## Abstract

**Background:**

Ethiopia is embarking upon a ground-breaking plan to address the high levels of unmet need for mental health care by scaling up mental health care integrated within primary care. Health system governance is expected to impact critically upon the success or otherwise of this important initiative. The objective of the study was to explore the barriers, facilitators and potential strategies to promote good health system governance in relation to scale-up of mental health care in Ethiopia.

**Methods:**

A qualitative study was conducted using in-depth interviews. Key informants were selected purposively from national and regional level policy-makers, planners and service developers (n = 7) and district health office administrators and facility heads (n = 10) from a district in southern Ethiopia where a demonstration project to integrate mental health into primary care is underway. Topic guide development and analysis of transcripts were guided by an established framework for assessing health system governance, adapted for the Ethiopian context.

**Results:**

From the perspective of respondents, particular strengths of health system governance in Ethiopia included the presence of high level government support, the existence of a National Mental Health Strategy and the focus on integration of mental health care into primary care to improve the responsiveness of the health system. However, both national and district level respondents expressed concerns about low baseline awareness about mental health care planning, the presence of stigmatising attitudes, the level of transparency about planning decisions, limited leadership for mental health, lack of co-ordination of mental health planning, unreliable supplies of medication, inadequate health management information system indicators for monitoring implementation, unsustainable models for specialist mental health professional involvement in supervision and mentoring of primary care staff, lack of community mobilisation for mental health and low levels of empowerment and knowledge undermining meaningful involvement of stakeholders in local mental health care planning.

**Conclusions:**

To support scale-up of mental health care in Ethiopia, there is a critical need to strengthen leadership and co-ordination at the national, regional, zonal and district levels, expand indicators for routine monitoring of mental healthcare, promote service user involvement and address widespread stigma and low mental health awareness.

**Electronic supplementary material:**

The online version of this article (doi:10.1186/s13033-017-0144-4) contains supplementary material, which is available to authorized users.

## Background

Integration of mental health into primary care services is the core recommendation of the World Health Organization (WHO) mental health Gap Action Programme (mhGAP) [1] in order to improve access to evidence-based mental health care in low- and middle-income countries (LMICs). However, although evidence is accruing for the efficacy of specific treatment packages for prioritized mental, neurological and substance use disorders in LMIC settings, the evidence base to support real-world effectiveness of implementation at scale is lacking [2].

### Systems thinking and health system governance

Expert consensus and the experience of those seeking to implement integrated primary mental health care indicate that health system factors are likely to be critical [3, 4], in keeping with lessons learned from roll out of other global health programmes [5]. The WHO has delineated the following interlinked ‘building blocks’ of the health system: service delivery, health workforce, information, medical products and technologies, financing and governance [6, 7]. Systematic evaluation is needed of the impact of health system components and processes on mental health care scale-up, with attention paid to the dynamic interactions between system components and their interface with the wider political and organisational context [7]. Strategies can then be developed to strengthen mental health systems across varied LMIC settings. Such ‘systems thinking’ approaches are in their infancy in the field of global mental health [8], particularly in relation to governance. Health system governance (HSG) has been defined by WHO as “*ensuring that strategic policy frameworks exist and are combined with effective oversight, coalition building, regulation, attention to system*-*design and accountability*” [6]. Good health system governance has been conceptualised as a fundamental requirement for optimal functioning of all other health system components [9] and, therefore, forms a critical focus of enquiry.

### Mental health context in Ethiopia

Ethiopia is a low-income country in sub-Saharan Africa with a population approaching 100 million people [10]. Specialist mental health professionals are scarce. There are approximately 60 psychiatrists practicing in the public sector, with most located in the capital city of Addis Ababa, although psychiatrist-led services have recently expanded to regional centres [11]. Masters level psychiatric practitioners and Bachelors degree level psychiatric nurses provide hospital-based mental health care across the country, but these services are limited to urban centres. As a consequence, the treatment gap for mental health care is very high, with an estimated 90% of people with severe mental illnesses (including schizophrenia and bipolar disorder) never receiving evidence-based care, and less than 1% receiving continuing care [12]. Rigorous epidemiological studies have shown that the burden of mental health problems in Ethiopia is high [12, 13], with untreated mental health conditions associated with premature mortality [14], disability [15], adverse economic impacts [16], stigma, discrimination and human rights abuses [17, 18]. In response, the Federal Ministry of Health in Ethiopia is embarking upon a ground-breaking programme to scale up access to mental health care using the WHO model of integration into primary care.

### Policy and health system context

Governance of the Ethiopian health system operates at multiple levels [19]. The Federal Ministry of Health provides central direction with the national level health policy and five yearly health care plans which integrate all health conditions [20]. Each of Ethiopia’s nine regions and the city administrations can exercise some autonomy in terms of implementation of health care plans, although are constrained to meet nationally set targets for health care delivery and health outcomes. At the lowest level of health care planning within the system, District Health Offices are responsible for supplying a range of services tailored to the specific needs of the population that they serve and are expected to lead health care planning from the grassroots, through ‘district-led planning’.

In the current five year plan, there is a target to expand mental health care to 100% of districts by 2020 and to increase the number and range of routinely collected health management information system indicators for mental health [19]. The National Mental Health Strategy of Ethiopia provides a more detailed framework for integration of mental health into the primary care system [21]. Ethiopia has no legislation concerning the provision of treatment to people with mental illness against their will and there is no specific legislation to protect the rights of people with mental health conditions in the wider society [22]. However, Ethiopia is a signatory to the United Nations Convention of the Rights of People with Disability which includes the rights of people with mental disability [23]. New social and community-based insurance initiatives aim to reduce the catastrophic costs associated with out-of-pocket expenditure for health care [24].

In collaboration with the WHO, mhGAP was piloted in Ethiopia across four regions [25], leading to a governmental commitment to scale up mental health care through in-service training of health centre-based primary care workers using mhGAP intervention guidelines [26] and integrating mental health into the packages of care delivered by community-based health extension workers [27]. Research-led programmes to implement and evaluate integrated mental health care are also underway in Ethiopia, including the Programme for Improving Mental health carE (PRIME) [28, 29], the Africa Focus on Intervention Research for Mental Health (AFFIRM) [30] and the ‘Emerging mental health systems in LMICs’ (Emerald) programme [31].

The goal of the Emerald programme is to generate evidence and capacity to strengthen health systems to scale up mental health care and thereby improve mental health outcomes in six LMICs: Ethiopia, India, Nepal, Nigeria, South Africa and Uganda [31]. Emerald focuses specifically on the health system functions of financing, service delivery, information and governance. Qualitative studies have been conducted in all Emerald countries to explore HSG in relation to the mental health system, with published findings available from Nigeria [32], South Africa [33] and Uganda [34]. In this paper we report findings from the qualitative study conducted in Ethiopia in order to address the following research questions:

- What are the institutional, contextual and health system governance barriers and facilitators to scaling up mental health care in the Ethiopian setting?
- What system level strategies may be employed to maximise the success of scale-up in Ethiopia?

## Methods

### Study design

A qualitative study was conducted using the HSG evaluation framework developed by Siddiqi and colleagues [35], adapted to include the inter-relationships with other health system components [9] and for the specific case of mental health care integration into primary care [33].

### Setting

Ethiopia is a low-income country located in the Horn of Africa, ranked 174th out of 188 countries in the Human Development Index [36]. As recommended [35], the evaluation of HSG was conducted at both the national/regional level and at the district level. The PRIME implementation site was selected as the district site because plans were being developed there to integrate mental health care into primary care [28, 29]. PRIME is operational in Sodo district of the Gurage Zone, Southern Nations, Nationalities and Peoples Region, located around 100 km from the capital city of Addis Ababa. The district has a population of approximately 160,000 people and is almost 90% rural and predominantly Orthodox Christian in religion [28]. At the time of the study there were eight primary care health centres (one operating as a public–private partnership), around 54 frontline health posts staffed by community health extension workers and no mental health services in the district.

### Sample

Key informants were selected purposively from the national/regional and district level. At the national/regional level, planners (n = 3) and mental health leaders involved in service development (n = 4) participated. At the district level, key informants were sought from planners (n = 2) and health facility managers (n = 8) located in Sodo district. Sampling at the national and regional level was constrained by the small number of people involved in mental health care policy-making and planning. Similarly at the district level, sampling was limited by the availability of managers and planners who had exposure to the concept of integration of mental healthcare into primary care. All those who were invited to participate accepted the invitation.

### Data collection

Semi-structured interviews were carried out using a topic guide derived from the adapted HSG evaluation framework [33]. See Additional file 1. This HSG framework seeks to ensure comprehensive coverage of all aspects of health system governance. The main domains covered by the topic guide were: strategic vision and rule of law, collaboration and participation, responsiveness and integration, equity and inclusiveness, effectiveness and efficiency, information, transparency and accountability, and ethics, considered in relation to health system financing, human resources, medication and technologies, information systems, infrastructure and service delivery.

The national level informant interviews were conducted face-to-face in English by CH and AA, apart from two interviews with regional participants that were conducted by email due to time constraints and geographical inaccessibility of the informants. The district level interviews were conducted in Amharic, the official language of Ethiopia, by Masters level research assistants who were trained in qualitative methods. The interviews were transcribed in Amharic and then translated into English, with discussion in the Ethiopia team to resolve uncertainties over translation.

### Data analysis

A framework approach to analysis was taken [37]. An initial coding framework prepared for cross-country application was adapted at baseline for contextual relevance and modified further as coding progressed. The framework is presented in Additional file 2. The Siddiqi framework provided the parent themes, but sub-themes and child themes were derived from the data and reflected the Ethiopian context for scale-up. Coding of transcripts was carried out by CH, TE and DA. The coding team met frequently to allow inductive adaptation of the coding framework, to clarify the scope of specific codes and to ensure consistent application of codes to the data. Open source software (Opencode 4.0 [38]) was used to facilitate the handling and analysis of the qualitative dataset. Once coding was complete, TE and DA extracted the coded data and summarised the findings by participant and by code/theme in a matrix. The participants were grouped according to type of informant. The data summaries were then compared across participants and participant groups.

The preliminary findings from the study were presented at a meeting, which was convened by the Federal Ministry of Health in collaboration with Emerald, in July 2015 in order to explore the system barriers to scale-up of mental health care in Ethiopia. Participants included the mental health focal persons and representatives from Regional Health Bureaus for the regions involved in scale up of mental health care in Ethiopia.

### Ethical considerations

Ethical approval was obtained from the Institutional Review Board of the College of Health Sciences, Addis Ababa University (Reference No. 074/13/Psy). Participation was voluntary and only took place after informed consent had been given. Care was taken in the presentation of quotations to ensure that respondents could not be identified.

## Results

The findings are presented in relation to the adapted framework for analysing HSG [33], stratified by national/regional and district level respondents where differences were apparent. A summary of the findings is presented in Table 1. Further supporting data for each theme are included in Additional file 3. The matrices used for analysis of the district level informants are included in Additional files 4 and 5.

**Table 1:** Applying a health system governance analysis framework to mental health in Ethiopia

	National level	District level
Rule of law		
Legal framework	No mental health legislation, but political commitment to develop legislation. Moral imperative to scale up even without legislation. Important role of family and community in safeguarding people with mental illness	
Regulation	Mechanisms exist to handle patient complaints, but limited regulation of clinical practice, no professional codes of conduct and proliferating cadres of mental health workers with unclear roles and responsibilities	
Strategic vision		
Policy	High level political support and momentum for change, with guiding framework of National Mental Health Strategy. Need for a national co-ordinating body for mental health scale up and engagement of planners at regional, zonal and district levels. Combining mental health with NCDs has potential opportunities and disadvantages	Importance of having a central policy to give legitimacy to implementation at the regional, zonal and district levels
Planning and co-ordination	Budget for scale-up committed by the Ministry of Health. Preliminary scale-up plan developed. However, poor co-ordination of activities, patchy capability and interest across the regions. Need for dedicated mental health co-ordinator at the regional level. Planning problems because programme is new	Essential to have mental health co-ordinator who takes responsibility for planning and implementing the service Need to build capacity in mental health planning
Leadership	Strong mental health leadership at highest levels but weak lower down in health system, with high turnover of staff. Perceived under-use of senior Ethiopian mental health professionals. Need to build leadership capacity of mental health professionals	Need for strong advocate for mental health at each administrative level of the health system
Participation and consensus		
Participatory decision-making	Wide consultation with stakeholders to develop National Mental Health Strategy, but less ongoing consultation for planning and implementation. Gap in co-ordination of national level stakeholders. Strength of existing fora for involvement of community representatives in health planning. However, low levels of community mental health awareness may undermine involvement. Need for communities to own the programme	Systems already exist for consultation and involvement of healthcare providers and community in service planning, but no experience applying to mental health care. For successful implementation, need active community involvement and engagement with multi-sectoral stakeholders
Service user and caregiver participation	Importance of participation recognized, but no real forum for involvement of service users in planning. No culture of service user involvement in national level health system planning. Stigmatising attitudes an important barrier	Interest and willingness to involve service users and caregivers, with recognition of their potential contribution. Need to empower service users and caregiver to strengthen involvement. Precedent of involvement of people with HIV/AIDS
Responsiveness and integration of care		
Prioritisation and meeting mental health needs	Mental illness perceived as neglected. Greater empowerment of service users required to ensure that services meet their needs. Vitally important to increase demand for services in order to increase the priority given	Improved awareness of the unmet need for care in people with mental illness since PRIME awareness-raising. Recognition of neglect of mental illness. Lack of parity between physical and mental health conditions that needs to be addressed
Integration at facility	Strong support for integration model as an effective way to improve access and reduce stigma. Supervision needed to supplement the brief training and to motivate primary care workers. Concern about feasibility and sustainability of current supervision framework which relies on psychiatrists	Strong support for integration model. Important to increase skills of primary care workers. Expected to improve job satisfaction. Potential barriers are additional workload, lack of space and negative attitudes of health workers. Concern about how competent health workers will feel after brief training
Integration in community	Community as pivotal to success. Inadequate engagement of community in mhGAP pilot associated with low demand. Network of community health workers and health volunteers offers great potential, but need to raise awareness in community	Familiar with community engagement and mobilization. Community can be part of a more holistic response to mental health needs e.g. tackling root causes such as poverty. Role in tracing defaulters and supporting re-engagement with care
Effectiveness and efficiency		
Financing	Money committed to scale-up, but budget needed for wider health system change as well as brief training. Proposed health insurance scheme will cover mental health and reduce out-of-pocket payments. Integrating mental health in primary care expected to make mental health care affordable. Problem of low demand for accurate financial forecasting	Integrated care important for making care affordable by reducing transport and time costs, but medication costs may yet be prohibitive. No experience with health insurance scheme to date
Human resources	Integration of mental health care promises efficiencies, but does not take away need to expand specialist mental health workers. Need for political support to expand role of mental health professionals from direct clinical work to include service co-ordination, supervision and mentoring. Focus on in-service training rather than pre-service perceived as a barrier to efficiency due to high turnover of staff and difficulties offering timely extra in-service training. Not feasible to rely on health volunteers for psychosocial interventions	Low baseline capacity of health workers in mental health limits ability to benefit from stand-alone short courses. Lack of incentives for those taking on mental health care. High turnover of staff. Need to target new recruits as only expect staff to stay for 2 or 3 years. Possibility of leveraging HEWs and HDAs promises efficiency and effectiveness (affordable by the community). Concern about willingness of community actors to work without financial incentives. Problem of over-burdening of health extension workers
Infrastructure and equipment	Medication supply critical to the success of implementation. Problems with medication availability during pilot but not insurmountable. Need to stimulate demand to allow forecasting. Need for decision-support materials in local languages	Major concerns about medication supply: frequent stock-outs and medications close to expiry date. Depend on private sector which adds to cost. Concern about availability of space to manage people with behavioural disturbance and to ensure privacy
Equity and inclusiveness		
Access	Government has prioritised equitable access to care. Health insurance has potential to improve access for the poor. However, limited information on extent to which implementation has led to equitable access to care and considered to be a future priority Cultural acceptability of available treatments and widespread stigma as potential barriers to accessing care	Accessibility expected to be improved by locally available care, but still concern about affordability of medication. Need to overcome low awareness and stigma
Stigma	Importance of stigma in impeding implementation and scale-up accepted by all. Anti-stigma campaigns supported by most. Service user involvement in antistigma campaigns considered to be essential. Caveat that might be better to focus on tackling discrimination rather than stigma	Familiar with anti-stigma campaigns (for HIV/AIDS) and receptive to involvement. Tackling stigma seen as essential for successful implementation. Supportive of service user and caregiver involvement in antistigma activities
Ethics		
Quality assurance	Considered important but difficult and beyond what is achievable in the early stages of scale-up. For other illnesses, new quality assurance frameworks (and performance indicators) disseminated by MoH, but mental health not included. Community consultation as an important mechanism for evaluating quality	Strong existing mechanisms for quality assurance on paper. Quality assurance not applied to mental health to date
Safeguards for ethical research	No major concerns. Rigorous ethical review in place but unclear whether can monitor the actual conduct of studies	Low level of awareness about procedures to ensure ethical conduct of research
Intelligence and information		
Monitoring and evaluation	Widely seen as challenging, with inadequate existing practice. The necessary information is not collected routinely. Need for better mental health HMIS indicators. New mental health indicators are proposed in the National Mental Health Strategy but not implemented. Means that unable to identify problems with implementation in a timely fashion and impedes planning	Very limited routinely collected mental health indicators. HMIS indicators for aspects of chronic care present for TB/HIV and could be collected for mental health. Well-established mechanisms for looking at aggregated HMIS and using for planning. Patient feedback obtained through service satisfaction forms and satisfaction surveys
Accountability and transparency	Reasonable faith in government accounting systems to minimize the risk of corruption. Question about accountability and transparency of decisions made by policy-makers and planners. Enforcement of systems considered weak. Difficult to access information about budgets and planning in the public domain	Robust systems for health facilities to be held accountable e.g. health facility boards. Transparency of appointing new heads questioned, although clear criteria on paper

*mhGAP* mental health Gap Action Programme, *PRIME* Programme for Improving Mental health carE, *HEWs* health extension workers, *HDA* health development army, *HMIS* health management information system, *TB* tuberculosis, *HIV* human immunodeficiency virus

### Strategic vision and rule of law

Almost all national/regional level respondents spoke about the positive government support for improving access to mental health care in Ethiopia.

*“I really think that there is a certain momentum around mental health in Ethiopia, I can see that. I’m not aware of other countries in this region that have made such a progress” (National level, ID6).*

Particular emphasis was given to the high levels of governmental support at the national level. The value of the National Mental Health Strategy in solidifying this support in the longer term was seen as vital. A district level respondent considered the Strategy essential to give districts a mandate to expand mental health care. The assignment of a mental health focal person to be situated within the non-communicable diseases (NCDs) section of the FMOH was seen as a positive development for some but an issue of concern for others. On the positive side, NCDs were considered to be a higher political priority than mental health so that mental health would benefit from the close linkage. On the other hand, some respondents were concerned that other NCDs would be prioritized over mental health. The financial commitment from the Ministry of Health to mental health care scale-up was viewed by most national level respondents as important evidence of governmental support and a strong facilitator to the success and sustainability of scale-up.

Most respondents at the national level endorsed the desirability of mental health legislation, but were divided on the extent to which it was a current priority or, indeed, a necessary prerequisite to scaling up mental health care. The need for legislation that was workable within the Ethiopia context was emphasised rather than importing Western models.

### Planning and co-ordination

Most national level respondents reported that there was little expertise for mental health care planning at all administrative levels in the health system (including regional health bureaus and district health offices) because of the newness of the programme and the low baseline level of mental health care provision. As a consequence, there was little experience in mental health care planning and it was difficult to predict demand for services. There was optimism amongst some respondents that most of these issues would improve as the scale-up progressed.

In view of the decentralized system of health care planning in Ethiopia, all respondents observed that national-level policies could only be implemented successfully with the ‘buy-in’ of the Regional Health Bureaus, which was currently lacking due to low awareness.

*“The district and zonal health office is at best ignorant about mental health services. Mental health is totally out of the planning and management” (National level, ID2).*

The experience gained to date had underlined the need for committed mental health care co-ordinators at each organisational level.

*“We were trying to use … persons who were assigned as focal persons at the Regional Health Bureau to help to coordinate activities in the health facilities … But these people have other duties… Somebody who can play that role would be very critical I think because this programme will improve or go down, depending on its success…” (National level, ID7).*

A co-ordinator would be able to ensure adequate budget allocation and reliable supplies of medication, to oversee the monitoring and evaluation activities and to lead awareness-raising in the community. Several district level respondents also spoke of the need for clear directives flowing from the Federal Ministry of Health downwards.

Some respondents felt that mental health professionals were best placed to lead service planning and co-ordination, but that this approach was limited by both the small numbers of specialists and their lack of training and experience in leadership roles. The need to strengthen mental health leadership among non-mental health professionals currently involved in the general planning of healthcare was also thought to be essential. Respondents reported a range of barriers to achieving this goal: the high turnover of staff in these positions, the difficulty in recruiting appropriately skilled individuals due to low salaries, the lack of interest in mental health issues and stigmatizing attitudes towards mental health.

### Participation and consensus

The process of development of the National Mental Health Strategy was endorsed as participatory by most national level respondents. However, this spirit of consultation and participation was reported to have been less prominent following the launch of the Strategy and during the shift to the next phase of national planning and implementation. In particular most respondents perceived there to be a lack of involvement of the community, service users and non-health sectors in the planning of mental health scale-up.

*“Decisions are usually made from top down, with very little opportunity for participatory approach” (National level, ID3).*

An important barrier to greater involvement of stakeholders in the planning process was the absence of any formalised structure to facilitate involvement, for example, in the form of a committee or designated body.

District level respondents had little experience with mental health care planning but highlighted the existing mechanisms to ensure participatory planning for health services more generally. One respondent spoke of the value of involvement of key stakeholders from outside of the health system in the planning process, for example the police or organisations involved in promoting livelihoods, so that they would then be more active in their support of implementation and allow multi-sectoral co-ordination. Community participation in planning new services was seen by almost all district level respondents as vital to the success of the endeavour.

*“People at the grass root level should be convinced on the prepared plan otherwise we cannot achieve our goal” (District level, ID10).*

When asked directly, most district level respondents appeared receptive to the involvement of service users and caregivers in service development, although not necessarily in the initial planning of the new mental health service. They drew from their experiences of working with people with HIV to develop and improve services but acknowledged that they had no experience of working collaboratively with people with mental health problems.

### Responsiveness and integration

#### Prioritisation and meeting mental health needs

At the national level, all respondents recognised a high unmet need for mental health care and none doubted the need to expand mental health care. However, some respondents anticipated that there would be low levels of awareness within the regional, zonal and district health administrations about the unmet mental health needs of their populations. Several respondents identified a need to improve the capacity of Regional Health Bureaus to identify the specific mental health needs of their populations and plan accordingly. The existing system of periodic client satisfaction surveys was identified as one approach to identifying unmet needs. Most respondents expressed confidence that planners would respond to increased demand for services.

District level respondents spoke similarly of the neglect of mental health care at every level of the system. They reported that their recognition of the unmet needs of people with mental illness had been improved through the awareness-raising carried out by the PRIME demonstration project. Low awareness and stigmatising attitudes were identified by all district level respondents as reasons for the low priority given to mental health care.

*“I think it is good, as we know mental health and people with this problem have been ignored. They have been suffering a lot and also didn’t get a proper care, and they have been referred to Addis Ababa and to other far places instead of getting treatment easily and get better like the other patients. In addition to this the society have negative attitude towards them…” (District level, ID17).*

#### Integration at facility level

There was strong support from almost all national level respondents for the plan to integrate mental health into primary care services. A potential barrier identified by a few respondents was the availability of adequate supervision for newly trained primary care workers.

*“I think based on the programme we are running, we cannot do it without supervision and mentoring…. So the training is short training, and people are just told about different illnesses and so they have to integrate it into their practice. And by mentoring and supervision, we are able to I think fill in that gap and also keep them motivated to work as part of their practice because these general health workers are not used to helping mentally ill people” (National level, ID7).*

Current models of centrally-based psychiatrists providing supervision and mentoring to newly trained primary care workers were seen as unsustainable due to the small numbers of available psychiatrists, their competing commitments and the cost of paying for their involvement. Making use of the expanding cadres of non-psychiatrist mental health specialists, for example psychiatric nurses and Masters level psychiatric practitioners, was raised as a workable alternative. However, there was some uncertainty about whether the existing system would be able to support a shift in the role of these mental health professionals, from clinician to that of trainer and supervisor.

*“Because currently, mainly their role is to just help patients who come to see them. But now this is an additional role. They’ll have to go out of their routine clinical practice to help in the training and supervision or mentoring” (National level, ID7).*

Another concern expressed by a national level respondent was about the acceptability of biomedical approaches to mental health care to most people in the community.

*“It could be the way, but I am not entirely convinced that people will show up to a health centre just because someone is there to give care; there are more complex issues why people do not access modern mental health care which need addressing” (National level, ID3).*

Almost all district level respondents thought that the needs of people with uncomplicated mental illness would be better served by receiving care in the primary care setting due to closer proximity, lower transport costs and lower time burden for patients and the accompanying caregivers.

*“I appreciate the idea of integrating mental health service to primary health care. This makes the service easily reachable to the society at large” (District level, ID10).*

District level respondents also anticipated that increasing treatment coverage for people with mental health problems would lead to reduced suffering and stigma. Timely intervention was expected to lead to better illness outcomes and to reduce the chance that mental health problems would develop into a more serious disorder.

One facilitating factor for introducing the new service model was a reported interest of primary healthcare workers to expand their areas of clinical competence. One respondent spoke of the enhanced job satisfaction that would result from being trained to deliver mental health care.

*“The other benefit is that the health professional will get satisfaction at least by giving a complete service. They also became competent and a better professional” (District level, ID12).*

Despite the positive attitude of most respondents, some respondents expressed concerns about the additional workload that would result from delivery of mental health care and the lack of time to see complex patients in the usual out-patient clinic. A few respondents anticipated that some health workers would be reluctant to get trained and that the additional workload could even reinforce negative attitudes towards people with mental health problems.

#### Integration in the community

The success of integrating mental health into primary care was reported by almost all respondents to be dependent on concomitant mobilization of the community. A relative lack of involvement of the community was seen as a limitation of current implementation activities, leading to low demand for mental health care at primary care facilities.

Many of the respondents detailed the focus given in the Ethiopian health system to community engagement and mobilisation in response to health issues. Respondents were quick to link the potential of this existing system to the plans for mental health care integration. Community-based health extension workers were seen as the natural gateway into the community, providing access to a network of health volunteers. Respondents were familiar with using these community structures to address other health conditions, although not mental health. From their experience with TB and HIV, for example, respondents reported that the community network could improve case-finding, raise community awareness, reduce stigma, engage traditional and religious healers and help track loss to follow up. The need to engage the community so that they own the programme was emphasised by all. Respondents also identified a potential role of the community-based health network in addressing the restraint of people with mental illness in their homes.

*“It is important, for example, they are in the community, they know all people in that sub*-*district and people who are tied in the households because they always go door to door, so, I think they are appropriate to identify and to create awareness in the society and bring them to the health facility if they can get simple training regarding mental health, so, it will be good if they can get training” (District level, ID17).*

Respondents noted that non-health structures within the community had a critical role to play in the successful implementation of integrated mental health care. As well as speaking to the critical role of traditional and faith healers and community leaders, many respondents spoke spontaneously about the role of engaging the non-health sector as part of the response to mental ill-health. This was noted both in relation to rehabilitation and to respond adequately to the root causes of disengagement from care and non-adherence to medication, most notably poverty.

*“When we come to mental health, the religious institutions have big roles…okay…they are the one(s) who can support us. …. I think we should collaborate with priests, edir [funeral group] leaders, and people who are working in agriculture and education sector.” (District level, ID 17).*

Respondents identified potential barriers to successful integration of mental health into existing community structures: the variable performance of different sub-districts and the difficulty in making interventions sustainable when relying on volunteers. An additional barrier articulated by one respondent was that health extension workers are known to have expertise in specific areas and that the community may not be convinced by the expansion of their role into mental health. However, other respondents referenced similar concerns about HIV, which had been shown to be surmountable. Some respondents were concerned about the capability of health extension workers and volunteers to grapple with mental health.

### Effectiveness and efficiency

#### Financing

Concern was expressed by a couple of national level respondents that the existing budget allocation focused narrowly on training for primary care workers and may not be sufficient to support the necessary health system changes to support scale-up of mental health care. However, there was appreciation from one respondent that the government had dedicated money towards scale-up activities.

At the district level, almost all respondents expressed concern about the prohibitive costs of out-of-pocket payments for medication needed on a long-term basis to treat mental illness.

*“I don’t think a person with mental health problem can afford the price of these medicines. We have to see in Ethiopian context…I think we have to give them freely. Mental health medicines are expensive. If it is given freely it should be supplied sustainably.” (District level, ID13).*

However, improving accessibility of mental health care through integration into primary care was seen by most as a positive intervention to decrease the financial burden on individuals. The planned roll-out of community-based health insurance and social insurance was welcomed.

#### Human resources

Making use of existing non-specialist health personnel to expand the reach of mental health care was seen as an efficient use of resources by most respondents. However, national level respondents were quick to point out that task sharing did not eliminate the need to expand specialist mental health professionals, as specialists would also be needed to ensure successful scale-up. Another concern was the efficiency of in-service training of primary care workers. One major barrier was the high level of turnover of primary care staff in rural areas, who were reported to often only stay in post for 2 or 3 years before moving to urban settings or to positions in non-governmental organisations. The drivers for high turnover were reported to be low salaries and limited opportunities for health workers to supplement their salaries, poor living conditions in rural areas and a lack of access to educational opportunities. To address this challenge, respondents argued for a focus on pre-service training.

*“What I would advocate for, which I think can really make a difference is reviewing the curriculum and having good integration of mental health in the curriculum because that saves even more money” (National level, ID5).*

Respondents spoke of some limitations in the ability of health facilities and district administrators to plan for recruitment and training of staff. Provision of in-service training in mental health care was reported to be dependent on external resources being made available and could not, therefore, be scheduled to meet the need as and when it arose.

#### Infrastructure and medication

The provision of reliable supplies of psychotropic medication was the main concern for most respondents. Numerous potential barriers were identified by respondents. Procurement systems for all medications were reported to be centralised, bureaucratic and lengthy. As a consequence, respondents reported stock-outs and supply of medications that were very close to their expiry date, obliging the primary healthcare facilities to purchase medications from private suppliers at a much higher cost. The difficulty in predicting demand compounded the problem.

District level respondents expressed concern about the lack of appropriate space for assessing and treating a person with mental illness. A particular concern was that people with acute behavioural disturbance would disrupt the clinic or block the clinic because they required a longer period for their consultation.

*“… it needs a well organised department, it shouldn’t be mixed with other types of services, and I don’t think there are health centers which have a separate room for mental health care service; but it will be good if we can have a quiet and confidential places for mental health patients” (District level, ID15).*

Respondents endorsed the need for up-to-date materials, for example treatment guidelines, to support the delivery of mental health care. It was suggested that such decision support materials would be more effective if they were translated into local languages.

### Equity and inclusiveness

#### Access to services

Government policies were reported to promote equitable access to all types of care, for example through the planned health insurance scheme, as well as broader policies to reduce poverty and increase education. Nonetheless, national respondents noted that there was a lack of information about the extent to which integrated mental health care was in fact accessed by vulnerable segments of society. One national level respondent acknowledged the importance of equity but saw this as something to be addressed later down the line, once the scale-up was underway.

#### Stigma

At the national level, stigma was recognised as a potent barrier to the successful scale-up of mental health care. Most respondents supported the idea of anti-stigma campaigns in an attempt to reduce stigma and had either participated in such events or expressed willingness to do so. District level respondents were clear that low levels of awareness and the presence of stigmatising attitudes were common in the community and a potential barrier for people with mental illness to access mental health care. The notion of community awareness-raising and anti-stigma campaigns was familiar to the district level health centre heads and administrators from their experiences with HIV/AIDS, although none of the respondents had been involved in mental health-related activities. All respondents expressed willingness to be involved and considered this to be part of the role of a primary care worker and necessary for the success of mental healthcare scale-up. Respondents valued the idea of involving people with mental illness who had recovered or their caregivers in awareness-raising activities and expressed the view that this would increase the impact on the community.

*“We can take the HIV anti*-*stigma as an example. We were successful because the patients expose themselves and teach the society share their experience. So patients and caregivers should participate on this anti stigma campaign” (District level, ID12).*

### Ethics and quality assurance

National level respondents acknowledged the importance of quality assurance but mostly considered this to be beyond what was achievable at this early stage in the implementation and scale-up of mental health care. The existence of quality assurance frameworks and performance indicators, disseminated by the Ministry of Health, was seen by most as a facilitating factor for future quality assurance in mental health care. Even though none of the quality assurance measures had been applied to mental health, some respondents were confident that this could be the case. Many district respondents spoke of the importance of getting feedback from the community in order to obtain a true sense of the adequacy of care.

### Intelligence and information

Deficits in the monitoring and evaluation of the scale-up of mental health care were seen as a critical barrier to scale-up by most national level respondents. Parallel systems of data collection were required because of the lack of mental health indicators included within the routine health surveillance systems. As a consequence it was difficult to obtain information about the functioning of the system and to detect problems in a timely fashion.

*“The information being gathered based on the routine information and data collection by the Ministry of Health does not help very much to get the right information… So I think the indicators that are useful, too, in helping the different level decision*-*makers are not available” (National level, ID7).*

Prioritisation of this area by the government was noted, through inclusion of new indicators in the National Mental health Strategy, although these had not been implemented. Respondents in the district spoke of the usefulness of the existing systems for monitoring and evaluation, covering every level within the health system.

*“The advantage of giving feedback is, (that) it will indicate activities which are not performed; for example the district administration can comment to the health centres about activities which haven’t be(en) done based on the data at hand. For example, there might be many cases in one administrative sub*-*district but they are working on some of them only, but if there are such type of processes, they will be forced or obligated to find the remaining cases and bring them to the health facilities” (District level, ID8).*

### Accountability and transparency

The existence of systems to ensure accountability was reported by most respondents, although some questioned the adequacy of their implementation. However, at the district level, many of the respondents felt themselves to be accountable to the community for planning decisions and service quality via regular public discussions and the feedback of the health boards affiliated to each health facility. Similarly the systems to ensure financial accountability were perceived to be robust, with concerns about accountability only raised when finance came from outside the government system, for example, from specific donors. District level respondents were unhappy about the seeming lack of accountability of the medication procurement agency for reportedly poor performance, but also spoke of the problems with accountability, for instance in terms of quality of medication, when they were forced to purchase medication from the private sector.

Respondents expressed less confidence in the transparency of decisions. Most respondents did not perceive that they could easily access information about budgets and planning decisions in the public domain. Other respondents countered that the difficulty was more one of collating the information rather than a problem of transparency. Questions were also raised about the transparency of systems for employing administrative and health facility heads.

## Discussion

In this qualitative study of key informants from Ethiopia, strengths and weaknesses of health system governance to support mental health care scale-up were identified through systematic application of an evaluation framework. Many of the governance challenges facing Ethiopia have been identified previously as barriers across LMICs in reports based on expert consensus [3]. A cross-country analysis of health system governance for the Emerald countries (Ethiopia, India, Nepal, Nigeria, South Africa, Uganda) has drawn out commonalities in challenges and opportunities [39]. Our study extends this previous work by identifying the areas of relative strength and weakness specific to the Ethiopian context which may usefully inform the ongoing efforts to scale-up mental health care.

The high level governmental commitment, development of a National Mental Health Strategy, expansion of specialist mental health workers, integration of mental health into health extension worker upgrade training, specification of targets in the health care plan and dedicated budget are indicative of strong strategic vision for mental health care scale-up in Ethiopia. These aspects of HSG have been found to be weak in evaluations of HSGs in other LMICs [9], including other Emerald study sites [32]. Ethiopia has benefited from a strong tradition of population level mental health research which has served to quantify the extent and burden of mental disorders within Ethiopia and strengthen arguments underpinning advocacy efforts [18]. Alongside this, the Toronto–Addis Ababa Psychiatry Project (TAAPP) has contributed to the expansion of psychiatrists within Ethiopia through support of an in-country training programme [40]. TAAPP focuses on broad training competencies, including the ‘psychiatrist as advocate’, which has helped to build up a critical mass of informed mental health professionals ready to engage with the Federal Ministry of Health. These factors, combined with committed and visionary leadership within the Ministry, mean that Ethiopia is in a substantially better position than 10 years ago to expand mental health care to the population.

Nonetheless, it was clear from this study that high level government commitment and a conducive policy context may not, by themselves, provide sufficient governance for successful scale-up. A recurring theme from respondents in our study was the need to enhance awareness, leadership, motivation and expertise in mental health care planning in order to successfully expand mental health care at the regional, zonal and district health administration levels. The current approach to scale-up of mental health care in Ethiopia involves working with the Regional Health Bureaus to train health workers from selected primary healthcare facilities across the region, thus bypassing the district-led planning system. With this approach, newly trained health workers return to their facilities but do not have system level support for delivery of mental healthcare. Such standalone mental health training interventions for primary care workers often have minimal impact on improving healthcare [41]. In the PRIME study in Ethiopia, brief training of primary care workers is being combined with engagement of the district health administration [29]. An initial half-day workshop to raise awareness about mental health led to the assignment of a mental health co-ordinator within the district health office. The PRIME team has provided ongoing technical support to the mental health co-ordinator with respect to mental health care planning, including medication procurement and training of trainers and supervisors for mental health care. The need to develop local leadership for mental health care accords with findings from the field of reproductive health, where managerial and leadership competence were identified as key system level factors distinguishing successful from non-successful implementation in primary care in rural Ethiopia [5]. In a recent systematic review of capacity-building initiatives for policy-makers and planners to strengthen mental health systems in LMICs [42], some promising approaches were identified. Although evaluation of the impact of such capacity-building approaches was limited, important ingredients for success appeared to include raising awareness of the public health relevance of mental health and tackling stigma, providing technical support within the context of an ongoing mentoring relationship and establishing networks for support and experience-sharing. Programmes to equip mental health specialists with the leadership skills needed to support system reform are also needed [43, 44]. In Ethiopia, there is a need to equip all cadres of mental health specialist with competencies in leadership and service planning to complement clinical skills.

The need for better co-ordination and involvement of stakeholders to allow for participatory planning was an important theme. At present there are limited mechanisms in Ethiopia for stakeholders to get involved in mental health care development and to have their voices heard. Low levels of mobilisation of service users within the country contributes to the problem and marks Ethiopia out compared to the other Emerald countries [32–34]. There are examples of successful involvement of service users in other LMICs in mental health system strengthening activities, including planning, service monitoring and advocacy, although the lack of rigorous evaluation of the impact of such initiatives is a barrier to more widespread uptake [45]. The Emerald project is piloting and evaluating approaches to encouraging more grassroots involvement of mental health service users to strengthen governance of integrated mental health care scale-up [46]. The importance of identifying leverage points within complex systems has emerged from analysis of successful mental health implementation projects in selected middle-income countries [8]. Enhanced service user involvement could be an important leverage point in the Ethiopian mental health system, with the potential to address a number of the main concerns arising in our analysis of governance: stigma, low awareness and demand, equitable access to care, quality of care, transparency and accountability of services.

Mental health integrated into primary care was perceived by all respondents to be more responsive and efficient than the existing centralised system of specialist mental health care. However, there was concern about how integrated care could be achieved in practice unless critical system level barriers were addressed. Chief among the barriers were low demand for mental health care (due to low awareness and stigma), lack of affordability of long-term care and inadequate supervision from mental health specialists. These factors may result in low uptake of care, expiry of medications (compounding supply chain obstacles), ineffectiveness of care and, ultimately, a lack of sustainability of services. Raising awareness about mental health conditions and their treatability in the community, combined with training community members to detect and refer possible cases, has been used successfully to increase uptake of mental health care in the PRIME implementation district [29]. This is a potentially scalable approach which could be integrated into health extension worker activities in order to support the national programme of mental health care scale-up. Given the brief nature of mhGAP training for primary care workers, ongoing supervision, mentoring and refresher training is essential for quality of care and to give primary care workers the confidence and impetus to deliver mental health care [3]. The shortage of mental health specialists in Ethiopia and their orientation towards hospital-based delivery of clinical care in hospitals means that regular supervision of newly trained primary care workers is not currently achievable. There is a need to expand the remit of mental health specialists to include supervision of primary care workers and to ensure that pre-service training equips specialists with the requisite skills. In the meantime, training the existing pool of non-specialist health worker supervisors in each district to also cover mental health care and combining this with telephone consultation with mental health specialists could help to address this important gap.

The inadequacy of existing mechanisms for the routine monitoring and evaluation of mental health care scale-up was considered to undermine several aspects of health system governance, including the responsiveness, effectiveness, efficiency, quality and equity of care. At present the Ethiopian health management information system (HMIS) only captures data about service utilisation on people with a ‘mental or behavioural disorder’ and ‘epilepsy’ which provides limited information to evaluate the service and support planning [47]. The optimal HMIS indicators required to monitor integrated mental health care in LMICs are not known. In a recent Delphi consensus exercise with experts from across six LMICs, a combination of indicators were endorsed to allow measurement of effective coverage (defined as “*the proportion of people in need of a service who gain the intended health benefit from that service*” [48]): items monitoring need for services, utilisation of care for specific mental health conditions, quality of care and financial protection [49]. An evaluation of the validity and utility of these selected indicators is underway in the Emerald countries.

### Strengths and limitations

Strengths of our study include the systematic and theoretically-driven approach to exploration of health system governance challenges. We included a range of relevant stakeholders, both at the national/regional level and the district level, thus encompassing perspectives from frontline managers as well as high level policy makers. The validity of our findings was enhanced by the opportunity to present our preliminary findings at the FMOH consultation meeting on mental health care scale-up. The study was limited by the relatively small number of interviews at all levels which reflected the limited pool of planners and managers with relevant expertise. As a consequence, theoretical saturation may not have been achieved. Service users and caregivers were not included in the current analysis because a separate study was conducted to investigate their involvement in aspects of mental health system strengthening, including governance [50].

## Conclusions

In order to support the scale-up of mental health care in Ethiopia, there is a critical need to strengthen leadership, co-ordination and capacity to plan mental health care at the national, regional, zonal and district levels, mobilise service users for greater involvement, expand monitoring and evaluation systems to capture mental health information and address widespread stigma and low awareness.

## Additional files

**Additional file 1.** Topic guides.

**Additional file 2.** Adapted analysis framework for health system governance in Ethiopia.

**Additional file 3.** Supporting quotations for each theme.

**Additional file 4.** Analysis matrix 1.

**Additional file 5.** Analysis matrix 2.

## Abbreviations

AFFIRM: Africa Focus on Intervention Research for Mental Health
Emerald: emerging mental health systems in low- and middle-income countries
HIV/AIDS: human immunodeficiency virus/acquired immunodeficiency syndrome
HSG: health system governance
LMICs: low- and middle-income countries
mhGAP: mental health Gap Action Programme
NCDs: non-communicable diseases
PHC: primary health care
PRIME: Programme for Improving Mental health carE
TAAPP: Toronto–Addis Ababa Psychiatry Programme
TB: tuberculosis
WHO: World Health Organisation
