# Supplementary material for: Health system governance to support scale up of mental health care in Ethiopia: a qualitative study
Source: Int J Ment Health Syst. 2017 Jun 8;11:38. doi: 10.1186/s13033-017-0144-4 (PMC5465569; doi:10.1186/s13033-017-0144-4)
Supplement: Supplementary file 1 — Additional file 1. Topic guides. [file 13033_2017_144_MOESM1_ESM.docx]

**Additional file 1: Topic guides**

**INTERVIEW SCHEDULE FOR NATIONAL LEVEL INFORMANTS**

**Aim of the interview**

The new National Mental Health Strategy of Ethiopia (Federal Ministry of Health) stipulates that mental health care should be integrated into primary health care. While a number of low- and middle-income countries have policies and legislation which support the integration of mental health care into primary health care to varying degrees, these are not sufficient to ensure transformation of the health care system towards integrated primary mental health care. In addition to the integration of packages of care for mental disorders using a task sharing (or task-shifting) approach, health systems strengthening is needed to support these integrated services.

The aim of this interview is to get your opinions on health system factors at the national level that you think might be barriers to or facilitate the implementation of Ethiopia’s mental health service integration plans.

**Research Question**

**What health system level processes are likely to aid or impede the implementation of mental health service integration plans in Ethiopia?**

|  |  |  |  |  |  |
| --- | --- | --- | --- | --- | --- |
| **1.      SYSTEMS FOR PLANNING AND MANAGEMENT** | | | |  |  |
| - 1. How do systems for mental health care planning and management operate in Ethiopia?   Probe for: | | | | | |
| 1. How decisions regarding mental health service planning are made, e.g., the need for additional specialist human resources/training of existing human resources to provide mental health care; | | | | | |
| 1. How are decisions regarding financing/budgeting for mental health services made, e.g. for both locally generated funds; donor funds | | | | | |
| 1.2 Challenges encountered in mental health care service planning and management | | | | | |
| 1.3 What are the possible measures/mechanisms that can facilitate mental health service planning? | | | | | |
| 1.4. Given that mental health care is a multi-sectoral endeavour, how does the MoH collaborate with other sectors at a national level in the development of mental health policies and plans? Probe for | | | | | |
| 1. Mechanisms/structures that facilitate this. If there aren’t existing mechanisms, probe for measures that can be put in place to facilitate this. | | | | | |
| 1.3. What is the extent of patient or caregiver participation in the planning and delivery of mental health policies and plans at a national level? Probe for | | | | | |
| 1. Are there any models of involving patients and/or caregivers in policy-making for mental health care in Ethiopia? 2. Probe for involvement in service development and implementation? 3. Probe for participating in monitoring service quality? 4. Probe for whether they think this could be achieved? What would be needed? 5. Probe for what they see as the challenges? 6. If there are no models for mental health care, what about for other health conditions? | | | | | |
|  |  |  |  |  |  |

| **2. INTELLIGENCE, INFORMATION** | |  |  |  |  |
| --- | --- | --- | --- | --- | --- |
| 2.1. How is information about the mental health system and mental health in the country used to inform the development of mental health policies, plans and the decision-making process? | | | | | |
| 2.2. How is implementation of mental health policies monitored?  If it is too early for them to say, probe for how other implementation of other health policies (e.g. HIV/AIDS policy) is implemented as a proxy for how implementation of mental health policy may be monitored. | | | | | |
|  |  |  |  |  |  |
| **3. ETHICS** |  |  |  |  |  |
| 3.1 What is the importance attached to better safeguards for mental health services e.g., protection of people being given treatment against their will? | | | | | |
| 3.2. In the case of research what is the importance attached to safeguarding participants from potentially unethical research, e.g., that ethical clearance is acquired before research and that informed consent procedures are adhered to? | | | | | |
| 3.3. Is there a policy and mechanism for promoting & enforcing safeguards for patients and enforcing codes of conduct for professional practice when it comes to mental health care? How could this be improved? | | | | | |
| 3.4. Similarly, for research are their mechanisms for protecting participants from potentially unethical research? | | | | | |
|  |  |  |  |  |  |
| **4. GOVERNANCE** |  |  |  |  |  |
| **4.1. Rule of law** |  |  |  |  |  |
| 4.1.1. Where are laws relevant to mental health initiated? | | | |  |  |
| 4.1.2. Are laws/regulations related to mental health service provision, infrastructure, technology, human resources, pharmaceuticals in place? | | | | | |
| 4.1.3. How are the laws translated into rules, regulations, and procedures? | | | | |  |
| 4.1.4. Does the MoH consult other line departments for laws/regulations pertaining to mental health? | | | | | |
| 4.1.5. What is the relationship of the MoH to the regulating bodies for mental health? | | | | | |
| 4.1.6. What is the capacity of MoH for contracting, regulating, accrediting, licensing of training programmes and mental health practitioners/organizationations/bodies that deliver mental health services? Probe for: | | | | | |
| 4.1.7. What procedures are in place for redressing grievances of (a) consumers,(b) contractors? | | | | | |
| 4.1.8. How are the relevant laws enforced? | |  |  |  |  |
| **4.2. Transparency** |  |  |  |  |  |
| 4.2.1 Is information about financial and administrative procedures in the MoH readily available? | | | | | |
| 4.2.2 How transparent is the process of resource allocation in the MoH? | | | | | |
| 4.2.3 Are there monitoring mechanisms in place to ensure transparency of decisions? | | | | | |
| 4.2.4 How does monitoring of mental health services occur? Who is responsible? | | | | | |
| 4.2.5 How are the district/facility managers appointed/transferred? | | | | |  |
| 4.2.6 What mechanisms are in place to ensure accountability of funds disbursed for mental health? | | | | | |
| **4.3. Responsiveness** |  |  |  |  |  |
| 4.3.1 How are funds for health allocated? | |  |  |  |  |
| 1. Probe for whether it is based on health burden, mortality, disability. | | | | |  |
| 1. Probe for how these mechanisms impact on the allocation of funds for mental health. | | | | | |
| 4.3.2 How does the MoH approach quality of health services and patient satisfaction? | | | | | |
| 1. Probe for mechanisms to monitor patient satisfaction generally and for mental health services more specifically | | | | | |
| 1. Probe for involvement of patients and caregivers in quality control. If none exist probe for openness to this possibility and possible suggestions on how this could operate. | | | | | |
| 4.3.3 How does the health system respond to regional/local priority health problems? | | | | | |
| 1. Probe for how the health system responds to regional/local mental health problems specifically. | | | | | |
| **4.4 Equity** |  |  |  |  |  |
| 4.4.1 Are there any social protection schemes in place to address financial barriers for the poor? How does this relate to mental health? | | | | | |
| 4.4.2 What policies are in place for identifying issues of equity in provision and financing of health services and rectifying them? | | | | | |
| 4.4.3 How equitable do you think is the allocation of public sector resources by states, provinces, districts equitable? | | | | | |
| **4.5. Effectiveness & efficiency** | | | | | |
| 4.5.1 What is the turnover/tenure of the Mental health leadership at the MoH? | | | | | |
| 4.5.2 What is the training, qualifications, experience of this leadership?  Probe about the challenges finding (and retaining) appropriately experienced people. | | | | |  |
| **4.6. Accountability** |  |  |  |  |  |
| 4.6.1 Are mechanisms for overseeing adherence to financial, administrative rules in place? | | | | | |
| 4.6.2 What evidence is present about the effective enforcement of accountability processes? | | | | | |
| **4.7. Stigma** |  |  |  |  |  |
| 4.7.1 What do you think is the proportion of burden of disease associated with mental health problems amongst people attending health facilities, and the community in general? | | | | | |
| 4.7.2 How important do you consider the integration of mental health care into primary health care service delivery? Probe for possible gains or disadvantages of integration. | | | | | |
| 4.7.3 What percent of the health care budget is currently allocated to mental health care? | | | | | |
| 1. Probe for whether they think this is appropriate. | | | | | |
| 1. If not appropriate probe for reasons as well as barriers to more funding and how attitudes towards mental illness may play a role. | | | | | |
| 4.7.4 Are you aware of any anti-stigma programmes? | | |  |  |  |
| 1. Probe for whether they have been involved/would like to be involved. | | | | |  |
| 1. Probe for whether they think service users/ health professionals should be involved in these activities. | | | | | |
|  |  |  |  |  |  |

**INTERVIEW SCHEDULE FOR ZONAL OR REGIONAL LEVEL MANAGERS/PHC CO-ORDINATORS**

**Aim of the interview**

The new National Mental Health Strategy of Ethiopia (Federal Ministry of Health) stipulates that mental health care should be integrated into primary health care. While a number of low- and middle-income countries have policies and legislation which support the integration of mental health care into primary health care to varying degrees, these are not sufficient to ensure transformation of the health care system towards integrated primary mental health care. In addition to the integration of packages of care for mental disorders using a task sharing (or task-shifting) approach, health systems strengthening is needed to support these integrated services.

The aim of this interview is to get your opinions on health system factors at the level of the zone (or region) that you think might be barriers to or facilitate the implementation of Ethiopia’s mental health service integration plans.

**Research Question**

**What health system level processes are likely to aid or impede the implementation of mental health service integration plans in Ethiopia?**

1. **AWARENESS OF THE IMPORTANCE OF MENTAL HEALTH AND REDUCED STIGMA**

1.1 How much of a burden do you think mental health problems are amongst people attending health centres? What about in the community?

1.2 In a setting like Ethiopia, how important do you think it is to have integration of mental health care into primary health care services?

a) Probe for possible advantages and disadvantages of integration.

1.3 How important do you think it is that primary health care staff are trained to deliver mental health care?

1. Probe for possible advantages and disadvantages of this training.

1.4 Are you aware of any anti-stigma programmes for mental health?

a) Probe for whether they have been involved/would like to be involved.

b) Probe for whether they think patients and/or caregivers should be involved in these activities.

c) Probe for whether they think health professionals should be involved in these activities

1. **HUMAN RESOURCE ISSUES**

2.1. How can the integration of mental health care be co-ordinated, at the zonal (or regional) level?

Probe for how much of a role a zonal [or regional] co-ordinator would have in the following activities:

1. Supporting people responsible for mental health co-ordination in the district health office.
2. Ensuring ongoing training of primary care staff in mental health care.
3. Monitoring the quality of services.
4. Ensuring reliable and timely supply of adequate medication.
5. Ensuring the timely appointment of specialist mental health professionals to support the primary care workers.
6. Organising awareness-raising and anti-stigma campaigns.
7. Any other roles?
   1. . What are the possible barriers/facilitating factors to having maximum coverage of staff trained in mental health guidelines?

Probe for how the following affect coverage:

1. Staff turn-over. Is there high staff turn-over? If so why? What measures can be taken to improve retention of staff?
2. Recruitment procedures. Are these procedures efficient (e.g., how long does it take to recruit and appoint new staff?). If not, what problems exist with recruitment procedures? What measures can be taken to improve these procedures?
3. Training procedures. Are there procedures in place that would ensure that new staff are quickly trained in mental health care. If not, how can these procedures be put in place? Are there procedures for refresher training? What measures can be taken to improve these procedures?

- 1. What are the possible barriers/facilitating factors for using **health extension workers** to help identify and/or provide psychosocial interventions?

Probe for the following:

1. Whether the role of **health extension workers** in mental health care would have official recognition.
2. Whether they would have time? Or sufficient skills?
3. What kind of support would they need? Are there systems in place to provide this support?
   1. What are the possible barriers/facilitating factors for using the **health development army** to help identify and/or provide psychosocial interventions (e.g. basic counselling)?

Probe for the following:

1. The sustainability of relying on health volunteers
2. Whether they would have sufficient skills?
3. What kind of support would they need? Are there systems in place to provide this support?
   1. What are the possible barriers/facilitating factors to having sufficient **specialist mental health professionals** to provide support (e.g. in terms of ongoing/refresher training, supervision, support and a referral service for more complex/treatment resistant cases)?

Probe for:

a) Staff turn-over. Is there high staff turn-over of specialist staff? If so why? What measures can be taken to improve retention of specialist staff?

b) Recruitment procedures. How much of a role does the district health office have in recruitment of specialist mental health professionals?

c) Training procedures. Are there procedures in place that would ensure that new specialist staff are trained to provide appropriate supervision and support? If not, how can these procedures be put in place? Who is responsible for ensuring this happens?

d) Attitudes of specialist staff towards task sharing. Are specialist staff supportive of diversifying their roles to provide training, supervision and support to non-specialist staff in mental health care?

**3. EQUIPMENT AND INFRASTRUCTURE FOR MHC**

3.1. How adequate is the supply of drugs/medications for mental health care in the zone (region)?

If not adequate, probe for how the following may affect drug supply

1. Drug policies. How do these impact on availability of drugs? Are psychotropic medications part of an essential drug list? Are these drugs made available free of charge to patients? What measures can be taken to improve drug policies?
2. Supply systems. How are drugs distributed to PHC clinics? What are the problems with the supply system? Are there buffer supplies/emergency stocks? What can be done to improve it?

3.2. What difficulties could there be with the supply of guidelines to treat mental disorders (and retaining these materials) in the clinics?

Probe for reasons for problems and possible procedures that can be put into place to overcome them.

3.3 To what extent do you think people with mental disorders will be able to speak privately to the health worker?

Probe:

Are there enough consultation rooms for people to be seen individually?

What issues could there be regarding confidentiality?

**4. SYSTEMS FOR PLANNING AND MANAGEMENT**

4.1. How do systems for planning and management operate? Probe for:

a) Where does healthcare planning take place? Zonal level? District level? Regional level? Is this the same for mental health care? Are decisions about different aspects of health care taken at different levels?

b) How can planning procedures for mental health care that are successful in one district be transferred to other districts (how can learning take place)?

4.2. Given that mental health care is a multi-sectoral endeavour, at a district level, how does the district health office collaborate with other sectors to tackle mental health problems and its determinants? Probe for

a) Mechanisms/structures that facilitate this. If there aren’t existing mechanisms, probe for measures that can be put in place to facilitate this.

5. **PATIENT PARTICIPATION**

5.1. What is the extent of patient or caregiver participation in the planning and delivery of mental health services?

5.2. How could patient or caregiver participation improve how mental health care is implemented in this zone / region?

Probe for:

1. How might patients and caregivers contribute to making the service development / implementation a success?
2. How could patients and caregivers be involved in monitoring quality / improving services?
3. How would you feel about working with patients and caregivers in this way?
4. What type of training might help you to work with patients and caregivers in this way?

**6. Monitoring and evaluation**

6.1 How can delivery of mental health care in primary care be monitored in a feasible and sustainable way?

Probe:

What kind of HMIS indicators would be useful?

6.2 Are there processes in place to give feedback to individual health centres about how well they are doing in terms of mental health care delivery?

Probe:

What could be done to improve this?

**7. Capacity Building**

7.1. We have been discussing many aspects of health system strengthening, especially focusing on mental health systems. Are there any parts of health system strengthening where you [or other people in the zonal office / regional health bureau] would value (additional) training?

*[Document spontaneous response]*

7.2. Then probe with the following for the capacity-building priorities within their organisation

**PRIORITIES FOR CAPACITY-BUILDING AT THE DISTRICT LEVEL**

|  | How important is it for your institution to build capacity in each of the following areas?  1 = irrelevant  2 = not a priority now  3 = important but not a priority  4 = a priority need  5 = an essential need | | | | |
| --- | --- | --- | --- | --- | --- |
|  | 1 | 2 | 3 | 4 | 5 |
| **Mental health policy, planning and programme development** | | | | | |
| Mental health policy development or policy review and re-formulation |  |  |  |  |  |
| Evidence-based mental health care planning |  |  |  |  |  |
| Mental health programme development |  |  |  |  |  |
| Planning for a system of mental health in primary care |  |  |  |  |  |
| Developing partnerships with patients for policy-making and service development |  |  |  |  |  |
| Human resources projection and cost calculation |  |  |  |  |  |

| **Mental health systems** | | | | | |
| --- | --- | --- | --- | --- | --- |
| Governance of mental health systems |  |  |  |  |  |
| Mental health system leadership |  |  |  |  |  |
| Mental health information systems |  |  |  |  |  |
| Mental health system communication |  |  |  |  |  |
| Mental health system advocacy strategies |  |  |  |  |  |
| **Mental health service implementation** | | | | | |
| Training for mental health workforce |  |  |  |  |  |
| Antistigma campaigns |  |  |  |  |  |
| Monitoring and evaluation of mental health services |  |  |  |  |  |
| Developing partnerships with patients to involve in quality control |  |  |  |  |  |
| Implementation of mental health services in post-conflict settings |  |  |  |  |  |
| Community-based approaches to mental health care |  |  |  |  |  |
| **Mental health research** | | | | | |
| Priority setting in mental health systems research |  |  |  |  |  |
| Conducting mental health needs assessments |  |  |  |  |  |
