# Supplementary material for: Health system governance to support scale up of mental health care in Ethiopia: a qualitative study
Source: Int J Ment Health Syst. 2017 Jun 8;11:38. doi: 10.1186/s13033-017-0144-4 (PMC5465569; doi:10.1186/s13033-017-0144-4)
Supplement: Supplementary file 2 — Additional file 2. Adapted analysis framework for health system governance in Ethiopia. [file 13033_2017_144_MOESM2_ESM.doc]

Additional file 2: Adapted analysis framework for health system governance in Ethiopia

| **Parent theme** | **Sub-themes** | **Child themes** | **Child themes** |
| --- | --- | --- | --- |
| **Rule of law** | Regulation of mental health care | Adequate level of regulation |  |
|  |  |  |  |
|  |  | Inadequate regulation | Handled through general regulatory body |
|  |  |  | Absence of codes of conduct |
|  | Absence of mental health law | Barriers to scale-up |  |
|  |  | Can be developed in parallel with scale-up |  |
|  |  | Priority |  |
| **Strategic vision** | Existence of mental health strategy | Strategy as facilitator for mental health care |  |
|  |  | Barriers to usefulness of Strategy |  |
|  |  | Consultation for Strategy |  |
|  | (4.1) Development and implementation of mental health plan | Barriers to development and implementation of plan | HR planning: specialist mental health professionals expansion |
|  |  |  | HR planning: specialist mental health professional role change |
|  |  |  | Weak monitoring of implementation |
|  |  |  | Weak HMIS for mental health |
|  |  |  | Feasibility and sustainability of supervision/mentoring framework |
|  |  |  | Challenges with regional co-ordination |
|  |  |  | Financial planning and forecasting (low demand) |
|  |  |  | Low awareness/commitment at lower level management |
|  |  |  | Low institutional memory |
|  |  | Facilitative factors for development and implementation of plan | Decentralisation of planning to regional health bureaus |
|  |  |  | Decentralisation of psychiatrists to regions (expansion of psychiatrists) |
|  |  |  | Supervision and mentoring framework |
|  |  |  | Political commitment at high levels |
|  | Leadership | Barriers | Limited number of people with adequate leadership capacity |
|  |  |  |  |
|  |  | Facilitators | Strong high level leadership |
|  |  |  | High commitment from mental health professionals |
| **Participation & consensus** | Coordination and consultation | Coordination & consultation barriers | Integration into general health policy/plan |
|  |  | Coordination & consultation facilitative factors | Plan to establish NIMH |
|  | Consultation/ involvement with service providers | Barriers to consultation with service providers |  |
|  |  |  |  |
|  |  | Facilitative factors for consultation with service providers |  |
|  |  |  |  |
|  | Consultation/ involvement with service users | Barriers to consultation with service users | Attitudes from leaders and administrators |
|  |  |  |  |
|  |  |  |  |
|  |  | Facilitative factors for consultation with service users | Precedent from other illness types |
|  |  |  | Empowerment of service users |
|  |  |  | Plan for NIMH |
|  | Consultation/ involvement with community | Facilitating consultation with community | Plan for NIMH |
|  |  | Barriers for consultation with community |  |
|  | Consultation with other sectors | Barriers to consultation with other sectors | Lack of co-ordination |
|  |  | Facilitative factors for consultation with other sectors | Plan for NIMH |
|  |  |  |  |
|  |  |  |  |
| **Responsiveness & integration of care** | Priority given to mental health | Competing priorities |  |
|  |  | Negative attitude/low awareness of importance |  |
|  |  |  |  |
|  | Integration at facility level | Negatives of integration | Lack of capacity in mental health care |
|  |  |  | Lack of time/busy workload |
|  |  |  | Negative attitude/not my job |
|  |  |  |  |
|  |  |  |  |
|  |  |  |  |
|  |  |  |  |

|  |  | Positives of integration | Improving other parts of general health care |
| --- | --- | --- | --- |
|  |  |  | Improving access |
|  |  |  | Benefit patients health |
|  |  |  | Benefit patients/ family’s finances and time |
|  |  |  | Health workers gaining additional skill |
|  | Mental illness burden & needs of users | Barriers to community based services | Low demand |
|  |  |  |  |
|  |  |  |  |
|  | Integration at community level | Barriers for community-based services | Low focus on community engagement |
|  |  | Facilitative factors for community based services | Existing community networks |
|  |  |  | Existing community engagement in health care |
|  |  |  |  |
|  |  |  |  |
| **Effectiveness & efficiency** | Financing | Financing barriers | Difficulty forecasting |
|  |  | Financing facilitative factors |  |
|  | Human resources capacity | Capacity barriers | Resistance/negative attitude |
|  |  |  | Staff turnover |
|  |  |  | Lack of incentive |
|  |  |  | Knowledge/competence gap for mental health |
|  |  |  | Lack of specialist (mental health) availability |
|  |  |  | PHC workers (facility-based) |
|  |  |  | Health extension workers |
|  |  |  | Health volunteers |
|  |  |  | Low level capacity to learn complex skills |
|  |  |  | Recruitment is long and inefficient |
|  |  |  | In-service training is not reliably available |
|  |  |  | Other training barriers |
|  |  |  |  |
|  |  |  |  |
|  |  |  |  |
|  |  | Capacity facilitative factors | More effective use of existing resources |
|  |  |  | Specialist mental health resources |
|  |  |  | PHC workers (facility-based) |
|  |  |  | Health extension workers |
|  |  |  | Health volunteer network |
|  |  |  | Experience of successful task sharing (e.g. HIV) |
|  |  |  |  |
|  |  |  |  |
|  |  |  |  |
|  | Infrastructure and equipment | Infrastructure barriers | Space barriers |
|  |  |  | Supply of drugs barriers |
|  |  |  | Supply of guidelines barriers |
|  |  | Infrastructure facilitative factors | Space facilitators |
|  |  |  | Supply of drugs facilitators |
|  |  |  | Supply of guidelines facilitators |
| **Equity & inclusiveness** | Access to services | Barriers to access | Paying for medication/investigations/ service |
|  |  |  | Distance |
|  |  |  |  |
|  |  | Facilitators to access |  |
|  |  |  |  |
|  |  |  |  |
|  | Stigma | Anti-stigma barriers |  |
|  |  | Anti-stigma facilitative factors |  |
| **Ethics** | Quality assurance | Barriers to service user satisfaction & quality assurance | Quality assurance not geared towards mental health issues |
|  |  |  | Quality assurance not practised at health centre level |
|  |  |  |  |
|  |  | Facilitative factors for service user satisfaction & quality assurance | Quality assurance frameworks from MoH |
|  |  |  |  |
|  |  |  |  |
|  |  |  |  |
|  | Safeguards against unethical research |  |  |
| **Intelligence & information** | M&E | M&E barriers | HMIS inadequate indicators for mental health |
|  |  |  | Low priority given/not being implemented |
|  |  |  |  |
|  |  | M&E facilitative factors |  |
|  |  |  |  |
| **Accountability** | Accountability barriers |  |  |
|  |  |  |  |
|  |  |  |  |
|  | Accountability facilitators | Robust systems to ensure accountability |  |
|  |  |  |  |
|  |  |  |  |
| **Transparency** | Transparency barriers | Inadequate consultation with stakeholders |  |
|  |  | Limited public access to factors affecting decision-making |  |
|  | Transparency facilitators | Fair systems |  |
|  |  |  |  |
