# Supplementary material for: Health system governance to support scale up of mental health care in Ethiopia: a qualitative study
Source: Int J Ment Health Syst. 2017 Jun 8;11:38. doi: 10.1186/s13033-017-0144-4 (PMC5465569; doi:10.1186/s13033-017-0144-4)
Supplement: Supplementary file 3 — Additional file 3. Supporting quotations for each theme. [file 13033_2017_144_MOESM3_ESM.docx]

**Additional File 3: supporting quotations for each theme**

Rule of law

So it's something we will have to work on in parallel and come with a proper mental health legislation to help also safeguard safety and these related issues…So I think like many other issues we're having, we have to also start doing this, but I'm not aware of any movement in that area. My feeling is the priority is now improving coverage. And maybe the need for that would be even felt more as it happens, and the magnitude of the issue and the context is better captured in the future.

National level (ID7)

Strategic vision

I think in the service in general has to be institutionalized. It should not in any way be dependent on individuals or a good will of individual. That's what I've witnessed in over the last two-three decades, because if one Minister of Health favouring mental health comes, then the mental issue becomes some - not a priority, but Number Three or Number Four agenda. If that person goes away, then so the priority issue goes down. … So what I am seeing very positive these days is that we have the document which is very important.

National level (ID5)

Yeah, I know that NCD, but there are also several competing programs…, now this Non-Communicable Diseases, the forefront are cardiovascular diseases, diabetes. These are issues that are very close to many policymakers. If you look at every person at the high level, he or she is affected in one way or the other with blood pressure, or high blood pressure, diabetes because of the living standard and many other issues. There are very many competing problems on the forefront, so at times, my worry is that mental health can also can be neglected….

If there is any kind of treatment program that goes according to the administrative setups we have it will be very practical, it is also good if it goes according to the government strategy.

District level (ID 16)

National level (ID5)

Planning and co-ordination

R: I think recently we are experiencing some good promise in terms of planning. However, in my opinion, much more coordination is needed, especially under the guidance of the Ministry of Health. *…* But the main issue in my opinion, the main advantage of having a coordinating body, first of all, it's to update each other on the progress that is done by the different actors, as I said, earlier. Because nobody's aware of what other people are doing and that's not a good thing.

National level (ID6)

The district and zonal health office is at best ignorant about mental health services. Mental health is totally out of the planning and management. Psychiatry nurses who return after completion of their training [sponsored by the region] are often subjected to neglect and disappointment. They are sometimes told there are no positions for them at the district level.

National level (ID2)

Well in some regions if you have excellent colleagues and very good champions for mental health, people with initiative and motivation who are willing to implement such programmes. And of course that makes a difference.

National level (ID6)

There should be one focal person to speak about this. You know there are administrative meetings, and individuals from each sector will be there. for example there is one person for TB , that person participate in many meetings representing his responsibility, whenever he is in the meeting he speak about his program so the members will be informed and create awareness and promotion. … If there is someone at least at the level of the district that may bring significant change in creating awareness, in relating the issue of the mental health program with other offices of the district.

National level (ID16)

I hope it might have feasible and sustainable monitoring if we can assign a focal person among professionals who are taking training now or if there is a person who can work accountably, that might help to evaluate. And also that person will be able to identify the gaps as well as unperformed activities. For example the other disease like HIV, TB... has a focal person each health care; for example if the implementation becomes poor, we are going to identify the sub-district which performs poorly and also the evaluation has been conducted based on that…

District level (ID8)

Leadership

I think generally, the attention at the Ministry of Health level is encouraging for mental health leaders in general in the country. … So that's one thing that's very positive about leadership on mental health in Ethiopia I think. But the challenge is we have the limited number of mental health professionals and I believe it's better to have mental health professionals as leaders for mental health. … So the problem would be about capacity and about widening perspectives and all this happens by building capacity and improving the skills and leadership and all that.

National level (ID7)

Participatory planning

Plans are not made by one person only instead they are prepared by many persons interaction. There is aggregate plan from the district prepared in consultation with the health center manager. The health center head will discuss with each case team members. Everybody can comment the plan and come to consensus before going to implementation.

District level (ID10)

In my opinion stakeholders should participate in mental health care service delivery. Mental health patients need psychological treatment and medical treatment from medical side. He may need societal acceptability, for example a mental health patient who is a civil servant needs acceptability from his colleagues. It is difficult to cure this person with drugs only. When there is a meeting to create awareness on mental health, people from police, court, finance and other area will be invited…In my opinion the problem is not having a focal person who feels ownership.

District level (ID12)

In my opinion stakeholders should participate in mental health care service delivery. Mental health patients need psychological treatment and medical treatment from medical side. He may need societal acceptability, for example a mental health patient who is a civil servant needs acceptability from his colleagues. It is difficult to cure this person with drugs only. When there is a meeting to create awareness on mental health, people from police, court, finance and other area will be invited…In my opinion the problem is not having a focal person who feels ownership.

District level (ID12)

[There is] a need to show mental health is relevant for other sectors. Ethiopia is not yet at that stage where various sectors recognize the fact that it is in the interest of the sector to take up mental health as its priority. That is the case even for the health sector. What needs to happen is a lot of advocacy and promotion.

National level (ID3)

I think that's an excellent initiative. I see that that is very difficult to implement that in Ethiopia. I think there's not that type of culture in Ethiopia so I don't know how workable it is.

National level (ID6)

R: But, you know I do believe, definitely, it is also our responsibility to help them organize but we are not doing a good job. We are not doing anything actually… Unless you involve the users, unless you involve the beneficiaries, how do you know? For me, it is very, very critical. And some day it is going to come, but it requires awareness, organization and stuff like that. I think it is very important.

National level (ID1)

There is only a nominal participation, the [mental health service] ‘user ‘association is at best promotional and no meaningful attempt is being taken by the ministry of health to engage them.

National level (ID3)

Actually, it is important. When we implement our plan we couldn’t say since you (patient) had this you should do this. To deliver quality service we have to make patients our center. To improve the quality of the service we prepare public forum. It is good to get comments from service users as long as we want to improve the service. I am not saying we do not have to involve them at all. I want to say we don’t have to involve them in the planning (at the beginning) but we have to involve them in the process of implementation. They may show us the right track.

District level (ID10)

…if that patient is treated well he will be witness and will publicize positively and propagate the good result of the program to the community, the treated-patients would spread out where they took the medicine and what type of program helped them restore their health. And in addition the participation of the patients or their caregivers will be a good source of constructive comments, on the both strength and weakness of the program.

District level (ID16)

Integration at facility level

The integration of mental health care into primary health care services is extremely important because it will improve access to mental health care of majority of the population. In addition it will probably be associated with lesser stigma than mental health care at mental hospitals.

National level (ID2)

Integration at the community level

… we shouldn’t leave everything to health extension workers because the community has to be properly empowered. After all, it should be the responsibility of the neighborhood, because if they see someone who is lately drinking with the binge drinking, for instance, it should be the sub-district administration elders who should come first and support that individual.

National level (ID5)

Effectiveness and efficiency

1. Financing

R: For this specific activity, which is just the small piece integrating mental health into primary healthcare. For this specific activity I didn’t feel that funding was a limiting factor.

I: Yes. But more generally for kind of the whole mental health systems from top to bottom going forward, do you think that's an issue?

R: I think it is an issue. But for that probably political commitment is a bigger issue.

National level (ID5)

Infrastructure and equipment

There is only one distributor in Ethiopia…, this is the only provider, but what we always receive is the undesirable, and very close to their expiring dates, and low quality, we prescribe or order the medicine but the public did not buy from us, because we don't have many of them, so they go to the private pharmacies. The private sector is a better provider in such cases.

District level (ID14)

Information

I think that component [monitoring and evaluation] is extremely weak if nonexistent. For the demonstration phase of mhGAP it was extremely difficult to gather the few data that we got. We developed some indicators that's not the part that worries me. But it's how you implement that and who is responsible for that, which is more concerning, I think.

National level (ID6)

But now what we are trying to do is to try to put some field indicators on the new management information system so that we can get some data on a regular basis. At least we cannot satisfy people who are requesting us information on mental health in every aspect, but we can at least say that the mental health program is functioning like this based on those few indicators, so they are sort of tracer indicators.

National level (ID4)

There is evaluation in some programs…okay… second there are programs which has quality evaluation, for example TB…okay…we use have the estimated number of service users (annually) in our plan; so the same can be applied to mental health…okay…so, if we can identify their number and then we can identify that how many of them have been treated and…uhh…in this way we can find out and how many of them have been properly treated can be also answered…okay…by looking at the service delivery system, the registration, reports we are doing follow ups, supports and also evaluations.

District level (ID9)

It is stated in a vague way, it is not clear about mental health indicators. We are not using HMIS indicators until they write it clearly. We are using our records. We have different disorders independently…. But it puts mental disorder as HMIS indicator, so, we can do nothing if we use this as an indicator.

District level (ID17)

TB and HIV have their own component in HMIS. HMIS doesn’t give specific indicators instead it gives general indicators. We made HMIS report for the some disease monthly. It may be case based and should be reported to the next level. In HIV there are OBC, orphan shelter clothing and others. If you take TB, we will report this much positives, this much cured outcomes, defaulters like that quarterly. If the disease needs long term treatment or lifelong treatment we have to check the status in certain time interval. There should be this kind of mechanism for mental health.

District level (ID10)

Accountability and transparency

Decisions are usually made from top down, with very little opportunity for a participatory approach and there is no process of accountability for reasonableness.

National level (ID3)
